# LEGO® Block Structures as a Sub-Kelvin Thermal Insulator

**DOI:** 10.1038/s41598-019-55616-7

**Published:** 2019-12-23

**Authors:** J. M. A. Chawner, A. T. Jones, M. T. Noble, G. R. Pickett, V. Tsepelin, D. E. Zmeev

**Affiliations:** Department of Physics, Lancaster University, Lancaster, LA1 4YB United Kingdom

**Keywords:** Applied physics, Techniques and instrumentation

## Abstract

We report measurements of the thermal conductance of a structure made from commercial Acrylonitrile Butadiene Styrene (ABS) modules, known as LEGO® blocks, in the temperature range from 70 mK to 1.8 K. A power law for the sample’s thermal conductivity *κ* = (8.7 ± 0.3) × 10^−5^ *T* ^1.75±0.02^ WK^−1^ m^−1^ was determined. We conclude that this ABS/void compound material provides better thermal isolation than well-known bulk insulator materials in the explored temperature range, whilst maintaining solid support. LEGO blocks represent a cheap and superlative alternative to materials such as Macor or Vespel. In our setup, <400 nW of power can heat an experimental area of 5 cm^2^ to over 1 K, without any significant change to the base temperature of the dilution refrigerator. This work suggests that custom-built modular materials with even better thermal performance could be readily and cheaply produced by 3D printing.

## Introduction

Low thermal conductivity materials are necessary for thermally isolating cryogenic components. Radiation shield spacers and support rods in dilution refrigerators are good examples of this. These components are useful for all cryogenics but especially for the current progression of quantum computing, which relies on isolated low temperatures for operation and coherence. Certain plastic materials, such as Vespel, have reasonably low thermal conductivities^1^, but large volumes can be costly. In this work, we show that a modular Acrylonitrile Butadiene Styrene (ABS) solid/void structure assembled from commercially available LEGO® blocks exhibit effective thermal conductivity even lower than industry-standard bulk materials, whilst offering good mechanical properties. The individual blocks readily allow affordable and repeatable large volume customization. Thermal conduction along the structure is difficult to predict from the properties of pure ABS material, since the internal thermal paths are complicated and include the solid-solid contact thermal resistance between the blocks. The results presented are characteristic of a modular ABS/void composite material constructed from typical LEGO elements.

## Results

**Figure 1:**
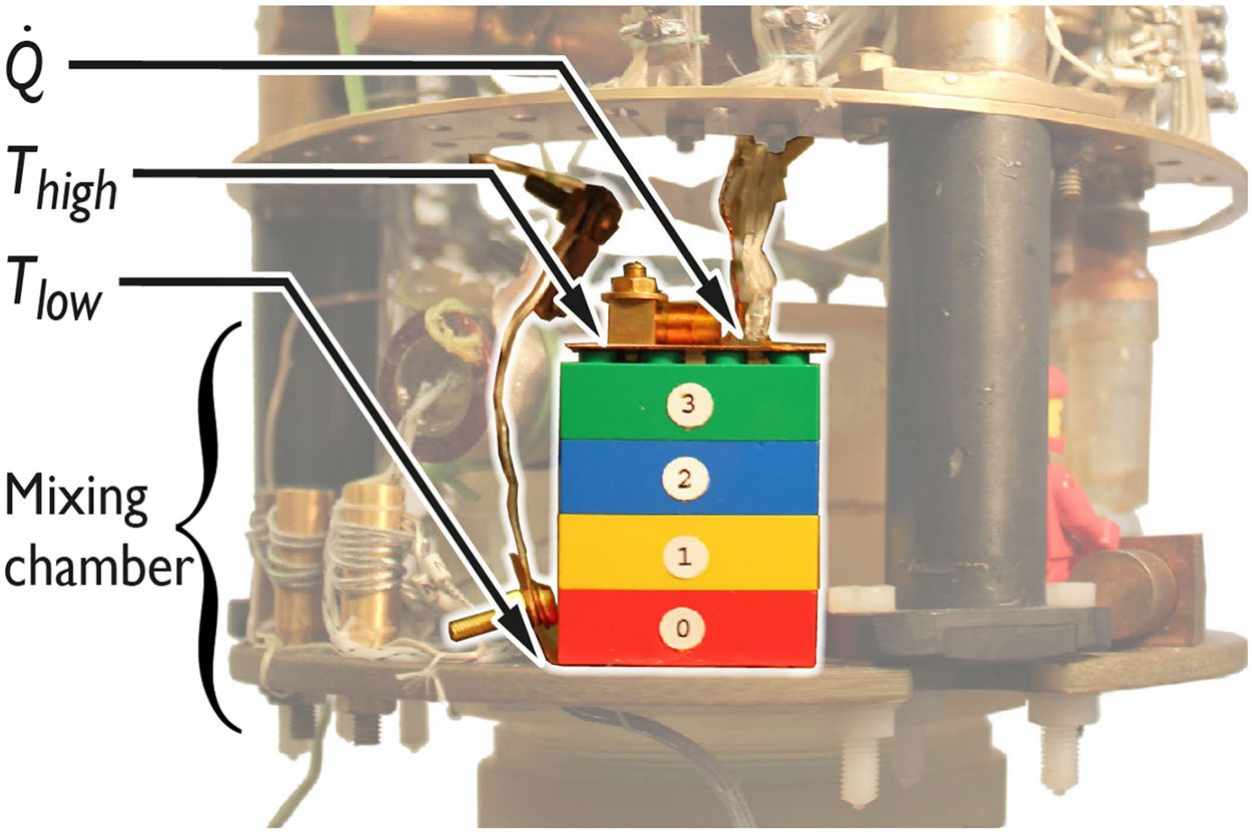
Experimental setup. Heat $\dot{Q}$ is applied via a 3 Ω manganin resistor and *T*_high_ is measured with a RuO_2_ resistance thermometer.

The experimental setup is shown in Fig. 1. We investigated a modular ABS structure comprising four standard LEGO blocks (Catalog No. 3001) stacked vertically and mounted in a Lancaster-built ^3^He/^4^He dilution refrigerator^2^. Since commercially available LEGO blocks are molded with a precision of *σ*_*x*_ ≈ 10 *μ*m^3^, it is very easy to reproduce structures accurately. The blocks were held together entirely by their interlocking geometry clamping power, with no added adhesive material. The stack had a total height Δ*x* = 40.2 mm, a footprint area of *a* = 502 mm^2^ and weighed 9.28 g. Copper-plate connections on the upper and lower ends of the structure were attached with the aid of vacuum grease to improve the thermal contact^4^. The lower Cu plate was connected thermally to the mixing chamber of the dilution unit, and on the upper Cu plate a 3 Ω Manganin wire heater and a calibrated RuO_2_ resistance thermometer were mounted.

After cooldown, the lower plate was held at *T*_low_ ≈ 4.5 mK for 9 days before the experiment was carried out. To measure the thermal conductance a constant heat level of $\dot{Q}$ was applied to the upper plate. After the upper plate temperature *T*_high_ stabilized a measurement was taken. A parasitic heat leak^5^ from the ABS structure (the slow leakage of heat from the ABS material itself) was measured to be ${\dot{Q}}_{0}=3.2\times {10}^{-10}\,{\rm{W}}$ (3.4 × 10^−11^ Wg^−1^), and was essentially constant over the time scale of the experiment.

For the thermal conductance of insulators at temperatures well below the Debye temperature, we can normally use the expression *κ* = *λT*^*n*^, where *κ* is the thermal conductance coefficient^6^. The constants *λ* and *n* can be determined by fitting the experimental data to the expression:

1 $$\lambda =\frac{\dot{Q}(n+1)\Delta x}{a({T}_{{\rm{high}}}^{n+1}-{T}_{{\rm{low}}}^{n+1})},$$

where *T*_high_ and *T*_low_ are respectively the high and low temperatures across the structure. This expression was obtained by integrating $\dot{Q}/a=-\,\lambda {T}^{n}dT/dx$ over the height of the sample.

Since in all our measurements *T*_high_ is much greater than *T*_low_, and *n* is found to be ~1.8, *T*_low_ can thus be safely neglected.

**Figure 2:**
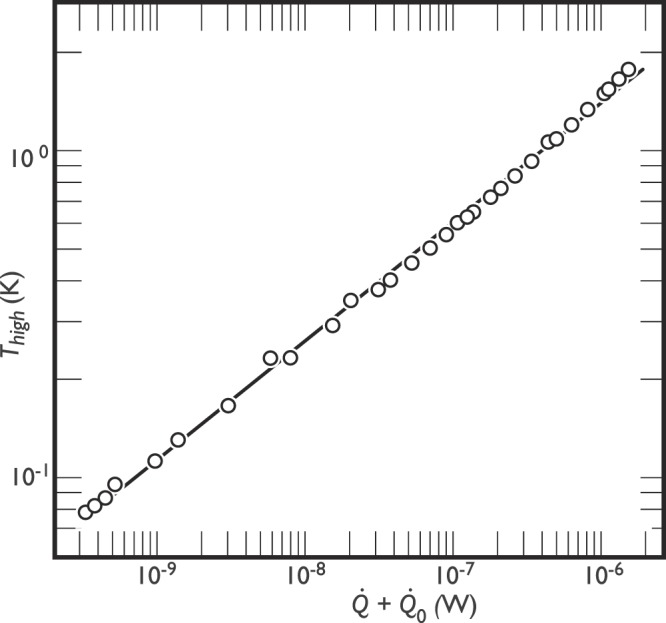
Vertical temperature gradient across the modular ABS structure, dependent on heat load $\dot{Q}$. A parasitic heat from the structure ${\dot{Q}}_{0}=3.2\times {10}^{-10}\,{\rm{W}}$ is added to our controlled heat $\dot{Q}$. The full-line fit to the data yields *n* = 1.75 (see text).

The measured results for $\dot{Q}$ versus *T*_high_ for the modular ABS structure are presented in Fig. 2. A least squares fit to our experimental data for the longitudinal thermal conductance yields:

2 $$\kappa =(8.7\pm 0.3)\times {10}^{-5}\,{T}^{1.75\pm 0.02}\,[{{\rm{WK}}}^{-1}\,{{\rm{m}}}^{-1}].$$

Thermal conductances in plastic materials at very low temperatures in general show *T*^*n*^ dependencies with *n* ranging between 1.7 and 2.4^6^, and our fit falls in this range. The thermal conductance of the extremely anisotropic modular ABS structure would clearly have a strong dependence on the axis of measurement.

Furthermore, and of importance in the current context, the modular ABS/void structure offers an order of magnitude lower thermal conductance than the best bulk thermal insulator, Macor^1^. The high level of insulation provided by the ABS structure most likely arises from the contact resistance between the individual LEGO blocks. As an illustration (taken from Fig. 1) the application of ≈400 nW of power to the top plate of the structure raises the top plate temperature to 1 K with no significant change in the bottom-plate (mixing chamber) temperature. For comparison, a Vespel-SP22 structure with the same footprint as the ABS modular structure would need to have a wall thickness of less than 300 *μ*m to achieve the same insulation^6^. A “No 3001” LEGO block has a minimum wall thickness of 1.20 mm, and was found to withstand ≈300 kg of load in a hydraulic press before failing. This demonstrates that it is mechanically robust despite the void space and will sustain any reasonable cryogenic experiment.

The thermal contraction of the ABS on cooling from room temperature to 4.2 K is 1.5%^7^ versus 0.6% for Vespel SP-22^6^. This could be important for certain applications, but for most applications, low thermal conductivity and cost are more important factors.

## Discussion

In this work, we have demonstrated that a modular Acrylonitrile Butadiene Styrene (ABS) structure assembled from LEGO blocks can provide a very effective thermal insulator at millikelvin temperatures. For a LEGO supported experiment requiring a 5 cm^2^ footprint, it is sufficient to supply less than 400 nW to achieve a temperature range from 100 mK to 1 K. This does not significantly change the temperature of the mixing chamber and therefore will not interfere with other experiments in the same dilution unit.

**Figure 3:**
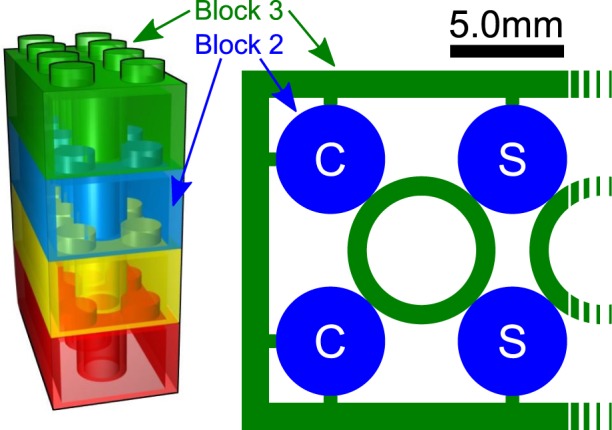
Left, a rendering of the modular ABS structure revealing the internal patterns of the LEGO blocks. Right, half of a cross section displaying internal block-to-block contact geometry, to scale. The entire shaded area representing block 3 rests on the upper surface of block 2. The 1.8 nm tall studs of block 2 provide secure interlocking to block 3, whilst minimising the contact surface area between blocks. Each connection has 4 ‘corner’ stud connections (labelled C), and 4 ‘side’ stud connections (labelled S).

There is no reason why thermal conductivity of bulk ABS should be very different from other polymer materials. Instead, we propose that the extremely low thermal conductivity of the structure can be attributed to the high resistance solid-solid connection between blocks, highlighted in Fig. 3.

The very beneficial properties of the composite vacuum/ABS structure measured here suggest that we can readily transfer the concept to 3D printed components. Already ABS is a popular base material for 3D printing. It would be straightforward to create complex cellular geometries with high strength, easy manipulation and low conductivity for use as a cryogenic insulator down to millikelvin temperatures and below. In this way, we could simultaneously tune the conductivities and mechanical strengths to suit the application, such as supporting the mixing plate of a dilution refrigerator based quantum computer. The motivation for this step is not simply one of the convenience offered from making complex structures via 3D printing, but one of conspicuous cost. In the current market, the price of a *single* sheet of Vespel of order 100 cm^2^ would cover the cost of the whole 3D printer setup needed for creating the ABS structures, which could be used repeatedly.

## Methods

The resistance of the calibrated RuO_2_ thermometer was measured using 4-point circuit with the Lakeshore 370 AC Resistance Bridge. The heat dissipated in the heater was controlled using a 4 point measurement as well. Bare NbTi wires, 40 cm long and 62 *μ*m diameter, were used for electrical connections to the thermometer and heater. The temperature of the dilute phase in the mixing chamber of the dilution refrigerator was measured using a vibrating wire resonator^8^. We changed the applied heat step-wise and waited for the temperatures to reasonably equilibrate before taking a measurement point (typically, 2 hours). The points presented in Fig. 2 were measured on both warming and cooling.

## Data Availability

All data used in this paper are available at 10.17635/lancaster/researchdata/328 including descriptions of the data sets.
